# Biochemical and *in silico* identification of the active site and the catalytic mechanism of the circadian deadenylase HESPERIN

**DOI:** 10.1002/2211-5463.13011

**Published:** 2022-03-29

**Authors:** Rafailia A. A. Beta, Athanasios Kyritsis, Veroniki Douka, Eirini Papanastasi, Marianna Rizouli, Demetres D. Leonidas, Dimitrios Vlachakis, Nikolaos A. A. Balatsos

**Affiliations:** ^1^ Department of Biochemistry and Biotechnology University of Thessaly Larissa Greece; ^2^ Genetics Laboratory Department of Biotechnology Agricultural University of Athens Greece; ^3^ Present address: Pulmonology Clinic University Hospital of Larissa Faculty of Medicine University of Thessaly Larissa Greece; ^4^ Present address: Department of Dermatology and Venereology Lausanne University Hospital (CHUV) University of Lausanne Lausanne Switzerland

**Keywords:** AtHESPERIN, circadian rhythms, deadenylation, mRNA decay, poly(A) tail

## Abstract

The 24‐h molecular clock is based on the stability of rhythmically expressed transcripts. The shortening of the poly(A) tail of mRNAs is often the first and rate‐limiting step that determines the lifespan of a mRNA and is catalyzed by deadenylases. Herein, we determine the catalytic site of Hesperin, a recently described circadian deadenylase in plants, using a modified site‐directed mutagenesis protocol and a custom vector, pATHRA. To explore the catalytic efficiency of AtHESPERIN, we investigated the effect of AMP and neomycin, and used molecular modeling simulations to propose a catalytic mechanism. Collectively, the biochemical and *in silico* results classify AtHESPERIN in the exonuclease–endonuclease–phosphatase deadenylase superfamily and contribute to the understanding of the intricate mechanisms of circadian mRNA turnover.

## Graphical abstract

Hesperin (AtHESPERIN) is the first circadian deadenylase discovered in plants. Herein, we determine its active site and provide information on the catalytic mechanism. We analyzed the kinetics of deadenylation reactions and modified Hanes–Woolf formalism together with application of molecular docking to study the effect of modulators on deadenylation reactions and oligomeric conformation of Hesperin. The results classify AtHESPERIN in the exonuclease–endonuclease–phosphatase deadenylase superfamily and contribute to the understanding of the intricate mechanisms of circadian mRNA turnover.

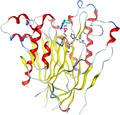

**Abbreviations**
DEPC: diethyl pyrocarbonate
EEP: exonuclease–endonuclease–phosphatase
GST: glutathione S‐transferase
MTG: monothioglycerol
SEC: size exclusion chromatography
SPC: single point charge

The circadian clock is based on interlocked feedback loops in transcription/translation that synchronize the timewise coordinated expression of genes. The stability of rhythmically expressed transcripts is critical for the maintenance of circadian rhythms [1]. mRNA concentration, at steady‐state levels, is an equilibrium of the rates of mRNA synthesis and decay [2]. An important structural component of eukaryotic mRNAs is the poly(A) tail at their 3′ end, which plays key roles in their stability, translational efficiency and degradation [1]. The poly(A) tail is a dynamic structure, the length of which varies over mRNA lifespan [1], and these variations follow the circadian clock [3, 4]. Nascent RNAs undergo poly(A) tail addition, where poly(A) binding proteins bind the tail and stabilize the transcripts while facilitating their export and translation [5]. Poly(A) binding proteins are also essential for poly(A) tail degradation, or deadenylation [6, 7], determining mRNA lifespan; shortening of the tail beyond a certain point triggers degradation and translational silencing [5, 8, 9]. Deadenylation is therefore a process that regulates the rates of transcription and translation [5]. Poly(A) shortening is catalyzed by a family of enzymes called deadenylases, which are classified into two groups: the DEDD and the exonuclease–endonuclease–phosphatase nucleases. DEDD nucleases are named after conserved Asp (D) and Glu (E) residues in their active site. EEP nucleases have conserved catalytic Asp, Glu and His residues [5, 10, 11], which are important for Mg^2+^ coordination and contribute to substrate positioning within the catalytic pocket [12].

Despite our understanding of deadenylases as regulators of gene expression, their role in the context of the circadian clock has yet to be explored in depth. Nocturnin was originally described as a deadenylase that follows rhythmic expression in mammals based mainly on a highly conserved endonuclease–exonuclease–phosphatase (EEP) domain present in the CCR4‐NOT and in early *in vitro* experiments [13, 14]. However, recent in‐depth biochemical studies revealed that Nocturnin is a phosphatase that converts its direct substrates NADP^+^ and NAPDH to NAD^+^ and NADH, respectively [15, 16]. *Hesperin* (*AtHESPERIN*) was identified as a homologue of Nocturnin in *Arabidopsis thaliana* and the first deadenylase with rhythmic expression in plants [17]. AtHESPERIN is oligomeric, most likely consisting of three identical subunits [17]. Bioinformatic analysis indicates that AtHESPERIN has a conserved EEP domain, displaying high sequence identity to other proteins that contain the EEP domain, such as the CNOT6L deadenylase and the earlier‐mentioned Nocturnin, and potential catalytic amino acids have been indicated [17].

Herein, to identify the catalytic mechanism of AtHESPERIN, we modified a site‐directed mutagenesis protocol and showed that E114, D287, D346 and H385 residues of Hesperin comprise the active site in line with the highly conserved catalytic motif among EEP deadenylases. In this direction and to improve the previously reported purification process of AtHESPERIN, we applied a mutagenesis protocol to generate an expression vector for *Escherichia coli*. Finally, molecular modeling simulations were used to explore the structural implications of Hesperin’s catalytic residues and their role in the catalytic mechanism. The results presented here show that AtHESPERIN is a member of the EEP superfamily of deadenylases. Molecular modeling indicates that AtHESPERIN follows the canonical/typical 3D structure of other EEP deadenylases.

## Materials and methods

### Site‐directed mutagenesis

Site‐directed mutagenesis was applied to synthesize all constructs described in this study. The pATHRA expression vector was used, and the E114A, D287A, D346A and H385A mutations of AtHESPERIN were introduced (Fig. S1). In brief, the DNA plasmid template was subjected to a two‐step PCR with mutagenic primers. Primer design was performed using the primerx tool (http://bioinformatics.org/primerx/), and reactions were performed with the KAPA HiFi HotStart ReadyMix PCR kit (KAPA Biosystems, Wilmington, MA, USA). The limitation of self‐annealing of the complementary primers in inhibiting PCR efficiency was circumvented by the separation of the reactions for the forward and reverse mutagenic primers to two distinct reaction tubes, and reactions were carried out according to the requirements of each mutagenesis. After the first PCR step (PCR_1_), reactions were mixed for a succeeding one (PCR_2_). Thermal profiles were designed according to the manufacturer's suggestions and were as follows: PCR_1_: 95 °C for 3 min, 98 °C for 20 s, 65 °C for 1 min and 72 °C for 8 min; PCR_2_: 95 °C for 3 min, 98 °C for 20 s, 60 °C for 1 min, 72 °C for 8 min, and a final extension step at 72 °C for 10 min. To remove the DNA plasmid template, which does not bear mutations from the sample, we subjected PCR products to digestion with Dpn1 (TaKaRa, Mountain View, CA, USA) following the manufacturer’s protocol. Finally, DNA was isolated by ethanol precipitation. Mutated DNA was reconstituted in nuclease‐free water and was used to transform competent Xl1‐Blue cells (Agilent Technologies, Santa Clara, CA, USA). Plasmid extraction was performed using the Nucleospin Plasmid kit (Macherey‐Nagel, Düren, Germany), and mutation insertion was verified by sequencing. To test whether this custom protocol is suitable for multiple mutations and substitutions, we applied it for the design of our modified expression vector (pATHRA). The primers used for these constructs are listed in Table 1.

**Table 1:** List of primers used for site‐directed mutagenesis. nt, nucleotides.

pATHRA	5′‐GATGATGCAGAGTTGTAGAACTAAACATGGGCCCCTGGAACAGAACTTCCAGGCTGCTGTGATGATGATGATGATGGCTG‐3′ (80 nt)
	5′‐CAGCCATCATCATCATCATCACAGCAGCCTGGAAGTTCTGTTCCAGGGGCCCATGTTTAGTTCTACAACTCTGCATCATC‐3′ (80 nt)
E114A	5′‐GATTTCTTTTGTCTGCAGGCGGTAGATGAGTACGATAGC‐3′ (39 nt)
	5′‐GCTATCGTACTCATCTACCGCCTGCAGACAAAAGAAATC‐3′ (39 nt)
D287A	5′‐CTACTAGCTGGCGCATTCAATTCAATTC‐3′ (28 nt)
	5′‐GAATTGAATTGAATGCGCCAGCTAGTAG‐3′ (28 nt)
D346A	5′‐CAAACACACTTGCGTACATCTTCATC‐3′ (26 nt)
	5′‐GATGAAGATGTACGCAAGTGTGTTTG‐3′ (26 nt)
H385A	5′‐CACCCGAGTGATGCATTACCTATAGG‐3′ (26 nt)
	5′‐CCTATAGGTAATGCATCACTCGGGTG‐3′ (26 nt)

### Construction of the pATHRA expression vector

pATHRA was constructed using the pET‐15b expression vector as a template. The thrombin recognition site of pET‐15b vector was mutated into the HRV 3C protease cleavage site. This required the substitution of eight consecutive amino acids, which was achieved by the design of the mutagenic primers (Table 1) to facilitate the substitution of 24 consecutive nucleotides in one reaction. The resulting pATHRA vector has restriction sites for BamHI and XhoI in its multiple cloning site. The N‐terminal 6× His‐tag is directly followed by the HRV 3C protease recognition motif. Reactions for mutagenesis were performed as described earlier. pATHRA may be compatible with the autoinduction method for recombinant protein overexpression from bacteria [18].

### Protein production and purification

pATHRA–HESP and pATHRA constructs for E114A, D287A, D346A and H385A AtHESPERIN mutants were used to transform competent BL21‐GOLD (DE3) cells (Agilent Technologies). A single colony was used to inoculate a 50‐mL preculture, which was incubated overnight at 37 °C with constant shaking at 210 r.p.m. Three liters of sterile LB Broth medium was inoculated with the preculture at 1/100 ratio, and cells were grown in the same conditions until induction (*A*_600_ = 0.6) with 1 mm IPTG and harvested (3 h postinduction) by centrifugation. Culture media were supplemented with 50 μg·mL^−1^ ampicillin. Purification conditions were similar for all proteins, unless otherwise indicated. Cells were thawed and resuspended in lysis buffer on ice. The optimal lysis buffer composition for each protein was as follows: 50 mm sodium citrate buffer (pH 6), supplemented with 0.2 m NaCl, 10% (v/v) glycerol, 0.1% (v/v) monothioglycerol (MTG), 1 mm PMSF and 1 mg·mL^−1^ lysozyme for E114A, D287A and D346A mutants, or 100 mm Tris–HCl buffer (pH 8), supplemented with 0.2 m NaCl, 10% (v/v) glycerol, 0.1% (v/v) MTG, 1 mm PMSF and 1 mg·mL^−1^ lysozyme for H385A, and 100 mm sodium phosphate buffer (pH 6), supplemented with 0.2 m NaCl, 10% (v/v) glycerol, 0.1% (v/v) MTG, 1 mm PMSF and 1 mg·mL^−1^ lysozyme for native AtHESPERIN preparations. The lysate was subjected to sonication followed by incubation with 50 U Benzonase (Sigma‐Aldrich, St. Louis, MO, USA) following the manufacturer’s instructions, centrifugation at 21 130 ***g*** and filtration through a 0.45‐µm filter (Sartorius, Göttingen, Germany). The clarified lysates were loaded onto a HisTrap HP column (GE Healthcare, Chicago, IL, USA) adapted on ÄKTA purifier. Binding buffers consisted of 50 mm sodium phosphate buffer (pH 6), supplemented with 0.2 m NaCl, 0.1 m imidazole and 0.1% (v/v) MTG (for E114A, D287A and D346A mutants), 100 mm Tris–HCl buffer (pH 8), supplemented with 0.2 m NaCl, 0.1 m imidazole and 0.1% (v/v) MTG (for H385A), or 50 mm sodium phosphate buffer (pH 6), supplemented with 0.2 m NaCl, 0.1 m imidazole and 0.1% (v/v) MTG (for native Hesperin). Elution buffer had the same formulation as the binding buffer supplemented with imidazole at 0.7 m final concentration. Proteins were eluted at 0.035 m imidazole gradient, analyzed by SDS/PAGE and dialyzed to 100 mm Tris–HCl buffer (pH 8), supplemented with 0.2 m NaCl and 0.1% (v/v) MTG. 6× His‐Tag was removed by overnight incubation with glutathione S‐transferase (GST)‐tagged HRV 3C protease, using a 1 : 20 ratio, at 4 °C. 3C‐Protease was removed from the sample using a Protino GST‐4B column (Macherey‐Nagel). GST buffer composition was as follows: 100 mm Tris–HCl buffer (pH 8), supplemented with 0.2 m NaCl and 0.1% (v/v) MTG, while elution buffer was supplemented with 20 mm reduced GSH. Finally, concentrated proteins were subjected to size exclusion chromatography (SEC) using a Tricorn Superose 12 10/300 GL column (GE Healthcare). The SEC run was performed with a 50 mm sodium citrate (pH 6), supplemented with 0.2 m NaCl and 0.1% (v/v) MTG. Protein purity was assessed by SDS/PAGE and the presence of uncleaved protein bearing a 6× His‐Tag by Western blot (SC‐8063; Santa Cruz). Fractions containing protein of high purity without the 6× His‐Tag were concentrated and dialyzed to reaction buffer (Buffer R), 50 mm sodium citrate (pH 6.5) or 50 mm sodium phosphate (pH 6.5), supplemented with 0.1 m NaCl, 2 mm MgCl_2_, 10% (v/v) glycerol in Amicon Ultra 4 or 15 centrifugal concentrator units (Merck, Darmstadt, Germany).

### Assays for poly(A) degradation

The enzymatic activity of recombinant wild‐type AtHESPERIN and its mutants was determined by a colorimetric assay and a fluorescence‐based assay.

#### Colorimetric assay

The colorimetric assay was performed as described previously [17, 19, 20, 21] with some modifications. In brief, poly(A) (Sigma‐Aldrich) was diluted in 100 μL Buffer R to final concentrations of 2–9 μg·mL^−1^. Substrate dilutions were mixed with 900 μL of 0.0012% (v/v) methylene blue/0.1 m MOPS–KOH solution and were incubated at 25 °C for 15 min to allow the binding of methylene blue dye into the substrate. Purified proteins were then added (*t* = 0), and measurements were taken over a time course of 5, 10, 15 and 30 min at the appropriate wavelength for poly(A)‐methylene blue mixture (628 nm) using a VIS‐UV 1600PC Spectrophotometer (VWR, Radnor, PA, USA). Measurements were used to calculate initial velocity values, and the results were plotted using grafit6 [22]. Enzyme activity reactions were carried out under constant enzyme concentration (0.4 μg·mL^−1^) and were performed in Buffer R (see earlier Protein production and purification section). Due to the relative light sensitivity of methylene blue, the reactions were carried out in the dark.

#### Fluorescence‐based assay

The measurement of enzymatic activity of AtHESPERIN using fluorescence was based on Maryati *et al*. [23] with some modifications. More specifically, a 17‐mer (5′‐CCUUUCCAAAAAAAAAΑ‐3′) oligonucleotide RNA substrate was synthesized (VBC Biotech, Vienna, Austria) bearing a 5′ Cy3 fluorescent group. Reactions were performed with 4 μm of the fluorescent substrate and enzyme concentration of 0.8 μm. Reactions were incubated at 25 °C and stopped at appropriate time points (1, 5, 10, 30 and 60 min) with the addition of 2× RNA sample buffer [95% (v/v) formamide, 5 mm EDTA, 0.025% (w/v) SDS] at 85 °C for 3 min. Reaction products were analyzed in denaturing 20% acrylamide : bisacrylamide/8 m urea–PAGE in a Mini‐PROTEAN Tetra cell system (Bio‐Rad, Hercules, CA, USA). All assays with Cy3‐labeled RNA were visualized using the Alliance 4 imager (Uvitec Cambridge, Cambridge, UK) unless otherwise indicated.

Inhibition by either AMP (Sigma‐Aldrich, St. Louis, MO, USA) or neomycin B sulfate (SERVA, Heidelberg, Germany) was tested with both assays at various concentrations. The Cy3‐labeled RNA assay in the presence of neomycin was visualized using ImageQuant Fluor imaging system [Cytiva (formerly GE Healthcare)]. All reactions were performed with the addition of 0.1 U RNasin (TaKaRa, Mountain View, CA, USA) and DEPC‐treated water.

### Kinetic analysis

Kinetic analysis was based on the colorimetric assay described in the previous paragraph. Analysis of kinetic data for allosteric enzymes was based on the Lineweaver–Burk [24] and the Hanes–Woolf formalisms [24, 25]. Fitting of the data was performed using the nonlinear regression software grafit6 [22].

### Native PAGE

The effect of inhibitors or poly(A) on AtHESPERIN’s conformation was assessed by native PAGE as described previously [17]. In brief, samples were prepared as in poly(A) degrading assays, where poly(A), AMP and neomycin concentrations were 50 × *K*_M_, and enzyme concentration was adjusted to [S]/10 (25 μg). The reactions were set up and incubated at 25 °C for 60 min; after incubation, they were immediately analyzed in a 7% PAGE under nondenaturing conditions for acidic proteins with appropriate molecular weight standards (MWND500; Sigma‐Aldrich). For the native PAGE analysis of AtHESPERIN in the presence of urea, 16 μg AtHESPERIN was incubated with increasing concentrations of urea, and then samples were analyzed in a 7% PAGE under nondenaturing conditions for acidic proteins with appropriate molecular weight standards. More specifically, BSA was used as an indicator of AtHESPERIN’s dimeric and trimeric conformations (BSA runs as two bands of 66 and 132 kDa), and urease was used to estimate the molar mass of higher‐order oligomers of AtHESPERIN [urease runs as a trimer (272 kDa) and a hexamer (545 kDa)].

### Western blotting

Protein samples before and after the SEC run were analyzed by SDS/PAGE and then transferred onto a Porablot poly(vinylidene difluoride) 0.22‐μm membrane (Macherey‐Nagel) in Towbin transfer buffer [26] supplemented with 0.1% SDS and 10% methanol using Yrdimes semidry Blotter (Wealtec, Sparks, NV, USA) following the manufacturer’s instructions. After transfer, the membrane was blocked with 5% skimmed milk in PBST for 1 h at room temperature and then incubated overnight at 4 °C with primary His‐tag antibody (SC‐8063; Santa Cruz), dissolved in blocking buffer (1 : 500). Primary antibody was probed using mouse immunoglobulin G horseradish peroxidase‐conjugated secondary antibody (Pierce, Waltham, MA, USA) diluted in blocking buffer (1 : 25 000), and visualization was performed with LumiSensor chemiluminescent HRP substrate kit (GenScript, Piscataway, NJ, USA).

### Homology modeling

The homology modeling of the AtHESPERIN deadenylase was performed using the Molecular Operating Environment Suite [24]. The 3NGQ RCSB (Research Collaboratory for Structural Bioinformatics) entry was used as a template, which is the crystal structure of the human CNOT6L nuclease domain [10]. The 3D models were subsequently energetically optimized in the CHARMM27 force field as it is implemented in the Gromacs Suite. Finally, all 3D models were assessed for their folding via procheck.

### Molecular docking

The docking module of MOE (molecular operating environment) was used for the holo complex of the AtHESPERIN model [27]. A fast Fourier transformation pipeline is used by MOE for the docking experiment. The overall score is influenced by the model’s packing, electrostatic, solvation and hydrophobic energies. Transient complexes of proteins are kept in a local database, and their contact propensities are statistically used for docking. The top hits of the docking experiment were energetically optimized using energy minimization pipelines to relieve the models from any residual geometrical strain. Finally, the Drugster suite was used to perform a final and rapid energy minimization step using amber99 force field [28], while solvating using an implicit generalized Born water model.

### Molecular dynamics

The interaction pattern and overall fold of the final complex of AMP and AtHESPERIN deadenylase model was subjected to exhaustive molecular dynamics simulations using the DrugOn suite [29]. Molecular dynamics simulations were executed in an explicitly single point charge water solvated periodic cube system. Counter‐ions were used as required to neutralize the molecular system. Each AMP/AtHESPERIN deadenylase model complex was subjected to 50 ns of molecular dynamics at 300 K and at 1‐fs step size. The molecular trajectory of each simulation was then imported into a local database for further analysis [29].

## Results and Discussion

### pATHRA vector and AtHESPERIN purification

**Fig. 1:**
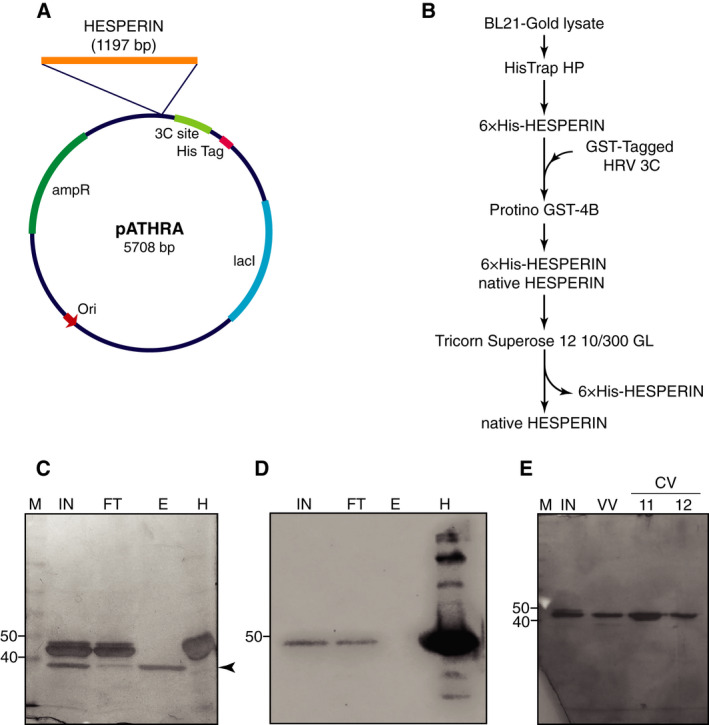
AtHESPERIN purification with pATHRA vector. (A) Cloning map of pATHRA. The insertion position and the length of the HESPERIN cDNA (orange bar) are indicated. (B–E) Purification of AtHESPERIN. Purification scheme of the recombinant AtHESPERIN (B). Digestion efficiency of GST‐tagged HRV 3C protease upon affinity chromatography was analyzed by SDS/PAGE (C) and western blotting using 6× His antibody to detect uncleaved AtHESPERIN (D). Both 6× His‐AtHESPERIN and cleaved AtHESPERIN are eluted from the GST column and subjected to a SEC step (E) where, uncleaved, 6× His‐AtHESPERIN is eluted in the void volume of the column. The arrowhead indicates the position of GST‐tagged HRV 3C protease; uncleaved AtHESPERIN is visualized with the use of primary His‐tag antibody. Numbers on the left of each gel indicate molecular size (kDa). CV, column volume; 11, 12: SEC fractions following the VV; E, elution; FT, GST‐4B column flow‐through; H, 6× HIS‐tagged AtHESPERIN used as a marker for western blot; IN, input; M, molecular mass marker; VV, void volume of SEC.

To elucidate the properties of AtHESPERIN (including molecular mass, oligomerization status and substrate preferences), we cloned the cDNA appropriately, and the protein has been purified with a protocol that includes affinity and SECs [17]. In our attempt to improve the purification protocol described earlier [17] and characterize AtHESPERIN in depth, we developed an expression vector based on pET15b, where the thrombin recognition site has been substituted by the HRV 3C protease recognition motif (Fig. 1A). We named the new vector pATHRA. Typically, thrombin is used to remove affinity tags from recombinant proteins. Yet, it has some disadvantages, such as low specificity and unpredictable cleavage of the recombinant protein of interest, inflexible digestion conditions, as well as difficult isolation from blood plasma and removal from the cleavage reaction [30]. To overcome the previous limitations, we used HRV 3C protease, among others, because it recognizes a motif of eight residues (LEVLFQ/GP) and is highly specific to this sequence, with high cleavage efficiency at 4 °C. Furthermore, it is commercially available and fused to a variety of affinity tags and easy‐to‐apply purification protocols. pATHRA was used to produce protein quantities in the milligram range, and purification was performed as described in the Materials and methods. After immobilized metal ion affinity chromatography and incubation of the eluted fractions with GST‐tagged HRV 3C protease, a second step to remove the GST‐tagged HRV 3C protease was performed (Fig. 1B,C). The resulting sample consists of a mixture of cleaved and uncleaved AtHESPERIN. The flow‐through fractions were analyzed by western blot to identify the fractions with uncleaved AtHESPERIN, as well as to estimate the relative efficiency of the cleavage reaction (Fig. 1C,D). Following this, the fractions of interest were concentrated and run through a Tricorn Superose 12 SEC column, where AtHESPERIN bearing the 6× His tag was removed (Fig. 1E). The efficiency of the purification protocol was estimated to be 0.6 mg recombinant protein per liter of bacterial cell culture, protein purity was evaluated by SDS/PAGE and its efficiency on retaining enzyme activity was assessed by deadenylation assays (see later).

### Active site of Hesperin

**Fig. 2:**
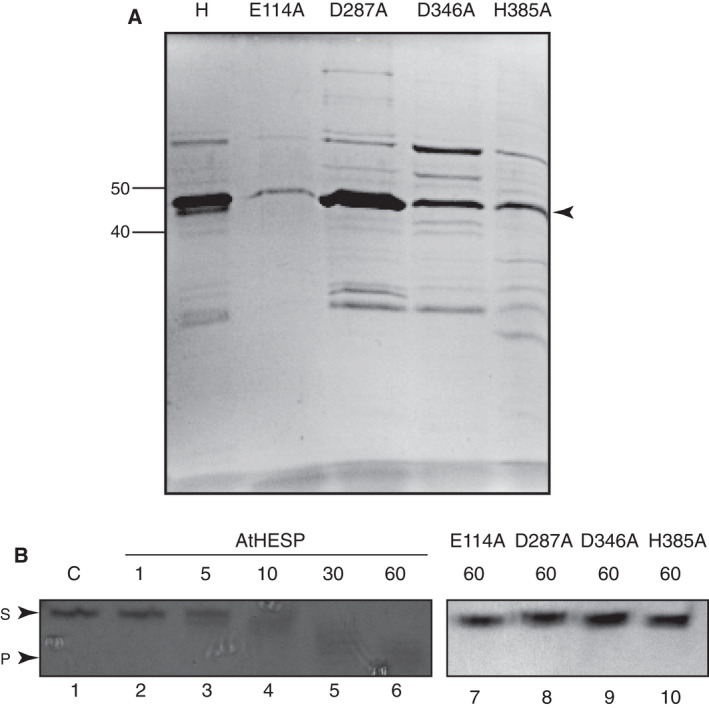
Identification of the active site of AtHESPERIN. (A) Purification of wild‐type AtHESPERIN (H) and E114A, D287A, D346A and H385A mutants. Proteins (10 μg each) were analyzed in a 10% gel. AtHESPERIN and its mutants differ in purity. The arrow on the left indicates the position of AtHESPERIN and mutants. Numbers on the left indicate the position of molecular mass markers. (B) Deadenylation activity of AtHESPERIN and mutants. Reaction time course in the presence of AtHESPERIN is shown in lanes 2–6. Reactions with E114A, D287A, D346A and H385A mutants are shown in lanes 7–10. All reactions were assessed with the fluorescence‐based assay. Reactions were performed in 50 mm sodium phosphate reaction buffer (pH 6.5) and were analyzed by urea–PAGE. The numbers on the top of the gels indicate reaction time in minutes. C, control reaction, where no enzyme is added; P, deadenylated product of the reaction; S, 5′ Cy3‐labeled fluorescent substrate.

The identification of catalytically important amino acids of AtHESPERIN was based on multiple‐sequence alignment of AtHESPERIN to other EEP deadenylases [17] (Fig. S1). Site‐directed mutagenesis of Glu114, Asp287, Asp346 and His385 to alanine was performed, and the purified mutant proteins (Fig. 2A) were further assayed for enzyme activity. To visualize and evaluate the shortening of adenosine tracts by AtHESPERIN, we performed a fluorescence‐based assay using a random oligoadenylated RNA sequence, instead of unlabeled poly(A) stretches or ^32^P‐labeled poly(A) substrates. To this end, a substrate bearing a 5′ Cy3 fluorescent group followed by a random ribonucleotide sequence and a 10‐adenosine 3′ tail was assayed against AtHESPERIN in a Na‐phosphate reaction buffer. Time‐course analysis showed faster migrating bands indicative of shorter reaction products because of the activity of the enzyme (Fig. 2B, lanes 1–6). Analysis of the reaction products revealed that E114A, D287A, D346A and H385 showed no significant deadenylation activity (Fig. 2B, lanes 7–10).

### Modulators of AtHESPERIN activity and oligomeric structure

**Fig. 3:**
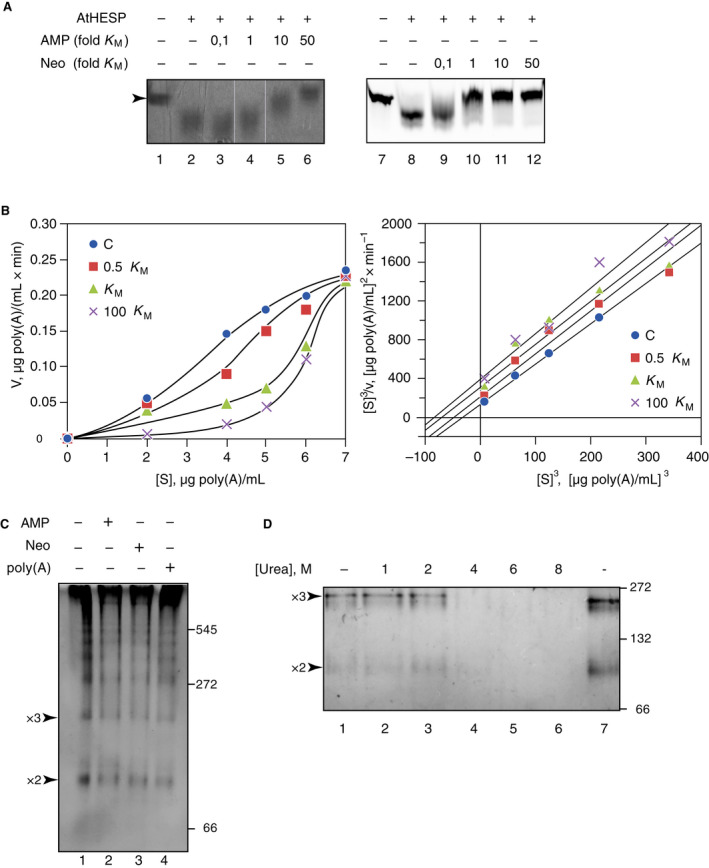
Effect of AMP and neomycin B on AtHESPERIN activity and oligomerization. (A) Deadenylation reactions by AtHESPERIN were performed in the presence of various concentrations of AMP or neomycin B (shown as fold *K*_Μ_ above the lanes). (B) Reaction rates of poly(A) degradation by AtHESPERIN in the presence of AMP quantified with a colorimetric assay. *v* − [S] plot of AtHESPERIN with increasing concentrations of AMP (left), and modified Hanes–Woolf plot indicating competitive inhibition (right). Plots were produced with the software grafit. (C, D) Native PAGE (7%) of AtHESPERIN incubated with poly(A) in the presence of AMP to assess changes in quaternary structure caused by the binding of the substrate or an inhibitory agent. Native PAGE of AtHESPERIN in the presence of AMP, neomycin B (C) or with increasing concentrations of urea (D). The arrows indicate positions of AtHESPERIN oligomers (2×: dimer ~90 kDa; 3×: trimer ~150 kDa).

To understand the catalytic mechanism of AtHESPERIN, we searched for small molecules that may modulate its catalytic behavior, such as AMP and neomycin. In this direction, the effect of 5′ AMP on AtHESPERIN activity was examined, because it is the product released by the activity of deadenylases [13, 31, 32]. Deadenylation reactions were performed in the presence of AMP at various *K*_M_ concentrations (*K*_M_ = 0.34 μg·mL^−1^). Analysis of the fluorescent reaction products on polyacrylamide gels showed that 5′ AMP efficiently reduced the activity of AtHESPERIN at high substrate concentrations (10–50 × *K*_M_), although it had a moderate effect at lower concentrations (0.1–1 × *K*_M_) (Fig. 3A, lanes 2–6).

Next, we examined the effect of neomycin B on AtHESPERIN’s activity, because it has been shown to inhibit Mg^+2^‐dependent deadenylases of the DEDD and EEP superfamilies, PARN [33] and CNOT6L [12], respectively. Reactions of 5′ Cy3‐RNA and AtHESPERIN were performed in the presence of increasing (fold *K*_M_) concentrations of neomycin B. Analysis of the reaction products on polyacrylamide gels showed that the presence of neomycin B in reactions catalyzed by AtHESPERIN produced a similar pattern, albeit less intense, as observed with AMP. The latter suggests that the two compounds inhibit the enzyme possibly through different mechanisms. These findings are summarized in Figure 3A showing that neomycin B inhibits AtHESPERIN’s deadenylase activity (lanes 7–10).

To analyze in depth the inhibitory profile of the small molecules, we further analyzed the kinetics of the inhibition of the reaction product, 5′ AMP. To this end, we performed deadenylation reactions with a colorimetric‐based assay. The results are depicted in Fig. 3B (left panel), as a *v* – [S] plot. Hesperin is an allosteric enzyme; therefore, plotting of kinetic data requires adaptations, as described previously [17]. More specifically, the Lineweaver–Burk equation requires 1/[S] to be raised to the power that will yield a straight line, that is, 1/v versus 1/[S]*^n^*, where *n* is the number of substrate binding sites [24]. However, Lineweaver–Burk plotting is problematic because it distorts the errors of the calculated *v* values. To overcome this limitation, we applied the Hanes–Woolf equation, which is a formalism of Lineweaver–Burk, where both sides of the equation are multiplied by [S] [24, 25]. Plotting our data as is (i.e. [S]/*v* versus [S]) again yields curved lines and for this reason, we raised [S] to the same power as in a Lineweaver–Burk plot to produce a straight line. Plotted data produced straight lines when [S] values were raised to the power of 3, in line with previous works [17]. Application of the Lineweaver–Burk and Hanes–Woolf formalisms on the colorimetric data showed that AMP is a competitive inhibitor of AtHESPERIN, as can be observed by the parallel lines (Fig. 3B, right panel). The calculated *K*_i_ value for AMP was 1.8 ± 0.2 μμ (0.625 ± 69.4 μg·mL^−1^).

AtHESPERIN is reported to form oligomers and most likely is active in a trimeric form [17]. We examined whether the inhibitory effect of AMP and neomycin B triggered any conformational changes of AtHESPERIN that might disrupt its active trimeric form. AtHESPERIN was incubated in assay conditions with 50 × *K*_M_ concentration of both AMP and neomycin B, and the reactions were analyzed in native PAGE (Fig. 3C). As controls, we included: (a) a reaction of AtHESPERIN without adding any of the examined molecules; and (b) a reaction where AtHESPERIN was incubated with poly(A), which would make for a band migration pattern representative of the oligomerization state of the active enzyme. All lanes had an identical band migration pattern, indicating that conformational change is unlikely to account for the loss of activity (Fig. 3C). Instead, the loss of activity might be attributed to the dislocation of catalytically important Mg^2+^ from the active site, as was the case for CNOT6L [12].

To further test whether the oligomeric conformation is transient and favored by hydrophobic interactions, we incubated AtHESPERIN with increasing concentrations of urea, ranging from 1 to 8 m. Native PAGE revealed a distinct migration pattern of AtHESPERIN corresponding to multiples of its molar mass. Observed trimeric structures persisted even in the presence of 4 m urea (Fig. 3D). When the concentration of urea increased to more than 4 m, the quaternary structure of AtHESPERIN is dissociated, yet the dimers or monomers were barely evident (Fig. 3D).

Taken together, the previous observations suggest that AtHESPERIN has a stable oligomeric conformation, mainly trimeric, not owing to weak hydrophobic interactions. AtHESPERIN together with PARN are the only described allosterically regulated deadenylases in plants and higher eukaryotes, respectively, thus far. Notably, *A. thaliana* PARN (AtPARN) shares significant sequence identity with the catalytic region of human PARN [34], yet a study to examine any allosteric behavior of *A. thaliana* PARN is still pending.

### Molecular modeling of AtHESPERIN and proposed catalytic mechanism

**Fig. 4:**
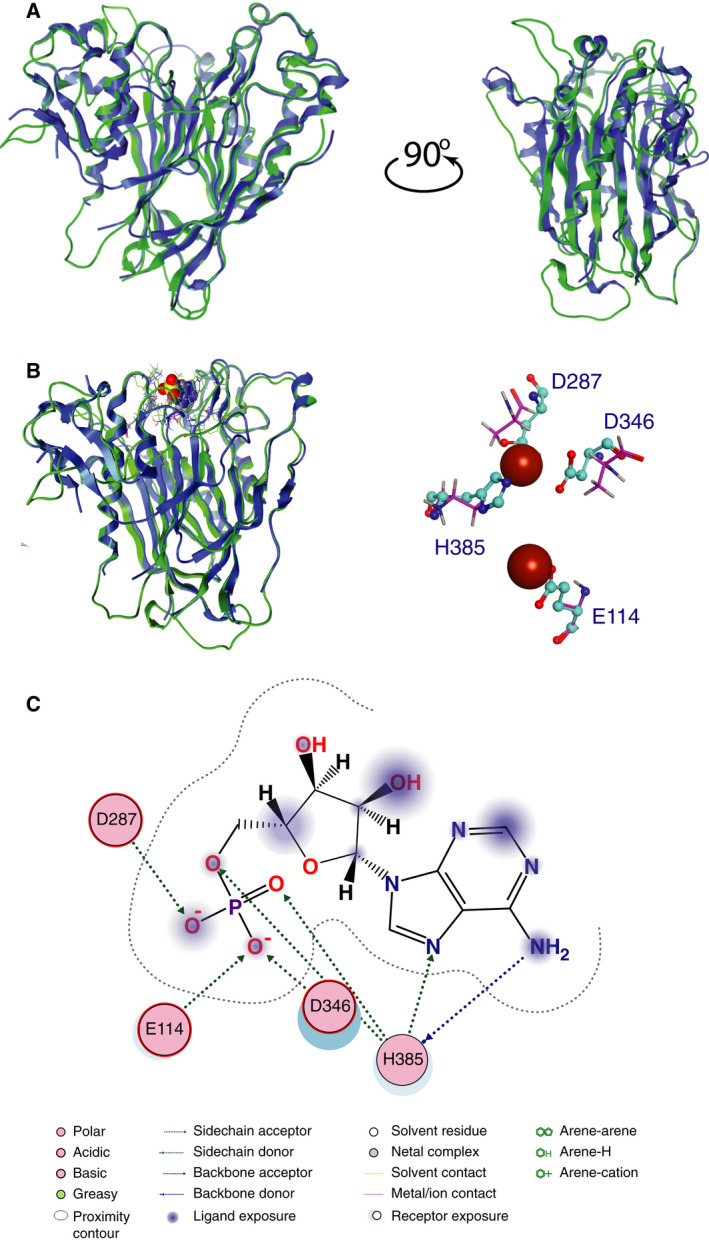
(A) Homology modeling of AtHESPERIN. The model is shown in blue ribbon representation, while the human CNOT6L template is in green. (B) Template and model of AtHESPERIN with the AMP bound at the catalytic site. The adopted ligand conformation, as well as the interaction pattern, is conserved, thus confirming the viability of the model. AMP is shown in a space‐filling model. A magnification of the site is shown in the right panel. Brown spheres define the conformational space of AMP. (C) Schematic representation showing the 2D interaction map of AMP to the AtHESPERIN model. The diagram was produced with ligplot [35].

The homology model of Hesperin (accession number UniProtKB: A8MS41) has conserved the 3D arrangement of the template structure by reproducing the beta‐sheet network and the main two alpha‐helices, flanking the catalytic site. The human CNOT6L (PDB: 3NGQ) [10] was used as a template for the homology modeling experiment in this study (see Materials and methods). The model of AtHESPERIN adopted the full conformation of its template structure (Fig. 4A). The core of the deadenylase is supported by a network of parallel and antiparallel beta sheets, whereas the outer domains of the enzyme consist of a series of alpha‐helices.

AMP was docked in the catalytic sites of both the template structure and the model of AtHESPERIN. The adopted AMP conformation, as well as the established interaction patterns, was conserved in the AtHESPERIN model, thus supporting the viability of the model (Fig. 4B).

Next, to further analyze the interaction between AMP and AtHESPERIN, as well the impact of the selected mutations, we had to identify the nature of the interaction. In particular, we wished to discriminate between direct hydrogen bonding and long nonbonded interactions. The ligplot analysis [35] revealed that the interacting residues E114, D287, D346 and H385 of AtHESPERIN establish direct interactions with AMP (Fig. 4C). Namely, for each pair of heavy atoms from the ligand set and the side‐chain receptor heavy atoms of all E114, D287, D346 and H385 residues, there was a tautomer combination in which one of the atoms was an H‐bond donor and the other an H‐bond acceptor. The short distance and optimal geometry orientation of all four interacting residues was quite favorable for the particular functional group, thus establishing a high‐energy H‐bond. The directionality of the hydrogen bond was calculated to set the minimum bound for what is considered to be a hydrogen bond under the given force field.

**Fig. 5:**
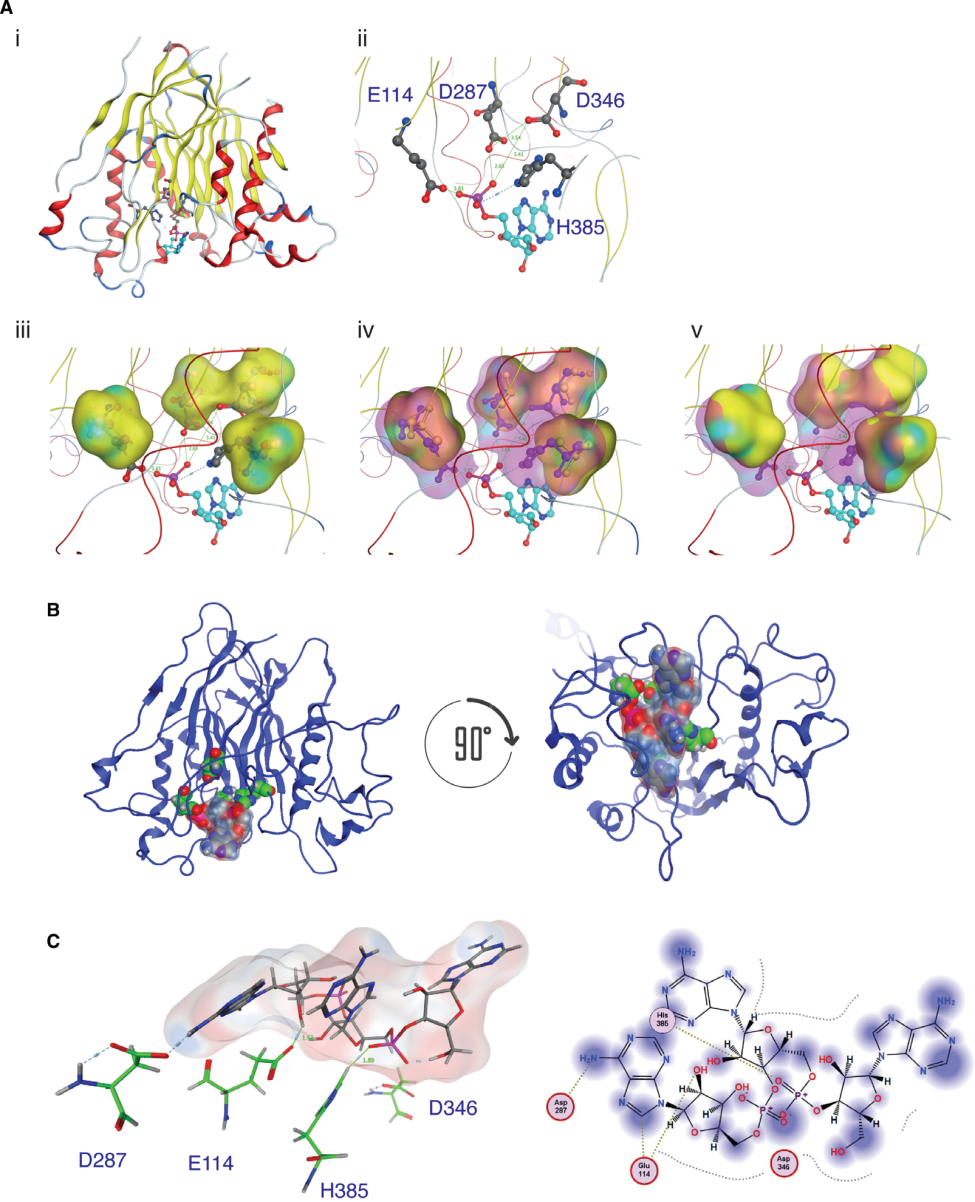
Molecular modeling of the loss of interaction and the proposed catalytic mechanism of AtHESPERIN. (A) Loss of interactions between AtHESPERIN and AMP upon mutations of the catalytic amino acids. (Ai) Model of AtHESPERIN with the catalytic amino acids (gray sticks/spheres) and AMP (adenosine: cyan/blue sticks and spheres). (Aii) Magnification of the catalytic site and docked AMP shown in (Ai). (Aiii) Conformational space of the alanine residues shown as yellow cloud superposed to the original E114, D287, D346 and H385 residues. (Aiv) Conformational space required by the original E114, D287, D346 and H385 residues and the alanine mutants shown as magenta cloud. (Av) Loss of the available conformational space shown as transparent yellow cloud. Sphere colors: cyan, AMP carbon atoms; gray, catalytic amino acid carbon atoms; red, oxygen; blue, nitrogen; magenta, phosphorus. (B) Proposed model for the catalytic mechanism of AtHESPERIN. The adenosine trinucleotide, A_3_ (blue‐red space‐filled model), docked in the catalytic site of AtHESPERIN (left panel) and tilted 90° backward on the horizontal axis (right panel). (Ci) The docked trinucleotide A_3_ (in blue–gray–red stick form in the surrounding conformational space) and the interactions established with the four catalytic residues of AtHESPERIN (in green stick form). (Cii) ligplot diagram showing the interactions between the four catalytic residues and A_3_ shown in (Ci).

The *in silico* mutation of any of the earlier‐mentioned residues to alanine resulted in the loss of interactions and significant changes in the physicochemical nature of the catalytic site and docking pattern. The loss of interactions between AtHESPERIN and AMP upon any combination of the E114A, D287A, D346A or H385A mutations is shown in Fig. 5A; clearly, replacing any of the E114, D287, D346 and H385 residues increases the conformational space of the catalytic site, and many key interactions to AMP are lost. Based on the previous, the homology model and AMP docking reveal structural similarities between AtHESPERIN and CNOT6L active sites.

Based on the previous data on the identification of catalytically important amino acids (Fig. 2) and considering that AtHESPERIN is an EEP deadenylase with preference for poly(A) [17], magnesium ions are necessary for activity [14], and the crystal structures of CNOT6L [10] and Nocturnin [36], we modeled the catalytic complex of the enzyme docked to a adenosine trinucleotide sequence (A_3_, Fig. 5B). A_3_ was capable of adopting only a single conformation in the docking experiment. Because only one conformation was acquired, we investigated the electrostatic surface (Fig. 5B) of the enzyme : substrate interface and the corresponding molecular interactions (Fig. 5C). The analysis showed that A_3_ interacts via H‐bonds with the four catalytic residues of AtHESPERIN. The data suggest a model where the two aspartic residues (D287 and D346) are in close proximity in the 3D conformational space of the catalytic site and initially coordinate the two Mg ions. Then, the imidazole ring of His385 acts as a base and attracts a proton from a water molecule. Consequently, the protonated histidine becomes activated for nucleophilic attack, while the neighboring Glu114 deprotonates the pentacoordinate intermediate, thus achieving hydrolytic cleavage of the phosphodiesteric bond. It is a well‐defined and coordinated mechanism, making the deadenylation reaction very dependent on the 3D conformation of the full enzyme that is absolutely essential to be conserved to coordinate the four catalytic residues in the exact optimal spatial positions in the proximity of the catalytic site of AtHESPERIN to achieve maximal efficacy.

## Conclusions

To characterize AtHESPERIN and determine its active site, we modified established site‐directed mutagenesis protocols and developed pATHRA, a custom expression vector. The modified protocol is efficient for both single and successive amino acid substitutions.

AtHESPERIN is an oligomeric EEP deadenylase and shares important features with other EEP members. Kinetic analysis showed inhibition by the reaction product (AMP) and neomycin B. Further, native PAGE does not indicate loss of AtHESPERIN’s quaternary structure, because the presence of poly(A), AMP and neomycin separately yield an identical band migration pattern (Fig. 3C) and may indicate the displacement of Mg^2+^ from the active site could account for loss of activity, as has been shown for CNOT6L [12].

We identified Glu114, Asp287, Asp346 and His385 as important residues for the catalytic activity of AtHESPERIN. Any substitution of each of these four residues abolished AtHESPERIN’s enzyme activity indicating their significance in the catalytic mechanism, by either coordinating Mg^2+^ or stabilizing substrate binding within the active site. Molecular modeling analyses confirmed that any of the earlier‐mentioned mutations result in the loss of interactions and consequently a thermodynamically less stable molecular system. As a result, these residues are essential to the function of AtHESPERIN. Collectively, our biochemical and *in silico* data suggest a cleavage mechanism for AtHESPERIN (Fig. 5B,C) characteristic of EEP deadenylases.

The biological role of this newly characterized enzyme was investigated in knockdown (T‐DNA) and overexpression mutant lines, which exhibit both developmental structural phenotypes and an altered response to stress conditions [14]. The data presented in this work provide essential further information toward the elucidation of its catalytic mechanism and overall 3D structure, as part of the biochemical characterization of the enzyme. This information is necessary to acquire targeted mutant lines and explore the biological role of AtHESPERIN.

## Conflict of interest

The authors declare no conflict of interest.

## Author contributions

NAAB conceived and designed the project; RAAB, AK, VD, EP and MR performed the experimental work and acquired the data; DV performed the molecular modeling analysis; RAAB, DDL, VD and NAAB analyzed the data and wrote the paper.

## Supporting information


**Fig. S1.** (A) Schematic representation of AtHESPERIN showing the conserved EEP nuclease domain in blue and the positions of the mutated amino acids. (B) Sequence map of AtHESPERIN. Blue characters represent the catalytic domain, and red characters in bold the mutated amino acids.

## Data Availability

The data that support the results of this study are available from the corresponding author upon reasonable request.
