# Supplementary material for: Biochemical and in silico identification of the active site and the catalytic mechanism of the circadian deadenylase HESPERIN
Source: FEBS Open Bio. 2022 Mar 29;12(5):1036–49. doi: 10.1002/2211-5463.13011 (PMC9063446; doi:10.1002/2211-5463.13011)
Supplement: Supplementary file 1 — Fig. S1. (A) Schematic representation of AtHESPERIN showing the conserved EEP nuclease domain in blue and the positions of the mutated amino acids. (B) Sequence map of AtHESPERIN. Blue characters represent the catalytic domain, and red characters in bold the mutated amino acids. [file FEB4-12-1036-s001.pdf]

```
        10         20         30         40         50         60
MFSSTTLHHL PRPNLLLPSS RVCRKVISRR MSTNPAIEPK VRKFESVEGV DIGSRNKSDG

        70         80         90        100        110        120
IRFRLVSYNI LAQVYVKSAL LPHSPPACLK WKARSHAILS VLKNLQADFF CLQEVDEYDS

       130        140        150        160        170        180
GFYRNNMDSL GYSGIYIQRT GQRKRDGCAI FYKPSCAELV TKERIEYNDL VDSIKADSVS

       190        200        210        220        230        240
CGSEQKIETS NEGKGDEKAK DSRKDSRDLN DPLVRLKRDC VGIMAAFRST SRFSISLSWQ

       250        260        270        280        290        300
THIFTGTLNW LMSLLKXKYL LSRXSSVQXG LISDEFECTP XLLLAGDXHF NSWGIWFIXT

       310        320        330        340        350        360
LVSXMAKPTE TIEEEEAPVP LSSVYEVTRG EPKFTNCTPG FTNTLDYIFI SPSDFIKPVS

       370        380        390
TLQLPEPDSP DVVGFLPNHH HPSDHLPIGA EFEIRRE
```

Figure S1
